# Unraveling the roles of endoplasmic reticulum-associated degradation in metabolic disorders

**DOI:** 10.3389/fendo.2023.1123769

**Published:** 2023-06-29

**Authors:** Hui Luo, Qibin Jiao, Chuanbin Shen, Chenyi Shao, Jinyan Xie, Yue Chen, Xinglin Feng, Xingwei Zhang

**Affiliations:** Department of Clinical Medicine, The Affiliated Hospital of Hangzhou Normal University, Hangzhou Normal University, Hangzhou, China

**Keywords:** ERAD, quality control, quantity control, proteasomal degradation, metabolic disorders

## Abstract

Misfolded proteins retained in the endoplasmic reticulum cause many human diseases. ER-associated degradation (ERAD) is one of the protein quality and quantity control system located at ER, which is responsible for translocating the misfolded proteins or properly folded but excess proteins out of the ER for proteasomal degradation. Recent studies have revealed that mice with ERAD deficiency in specific cell types exhibit impaired metabolism homeostasis and metabolic diseases. Here, we highlight the ERAD physiological functions in metabolic disorders in a substrate-dependent and cell type-specific manner.

## Introduction

Metabolism is a process of converting nutrients into energy to maintain cell growth and activity. Changes in external nutritional conditions and lifestyles have led to evolve a series of mechanisms for metabolic adaption. Metazoans can coordinate different hormones, metabolic tissues and organs to adapt to metabolic changes. The prevalence of overnutrition and sedentary lifestyles has led to global increase of metabolic disorders associated disease such as obesity, type 2 diabetes mellitus (T2DM), non-alcoholic fatty liver disease (NAFLD) and cardiovascular disease (1, 2).

Endoplasmic reticulum is the major compartment of protein folding, calcium storage and nutrient sensing (3). Lipid metabolism, protein balance, and calcium signaling processes controlled by the endoplasmic reticulum are closely related to metabolic disorders. Perturbation of ER homeostasis by cellular proteotoxicity, lipotoxicity and glucotoxicity can result in the accumulation of unfolded or misfolded proteins in the ER lumen and ER stress (4, 5). ER stress is commonly found in metabolic diseases including T2DM, insulin resistance, and NAFLD (6).

ERAD, unfolded protein response (UPR) and autophagy are the major protein quality and quantity control pathways to maintain ER homeostasis (7). Failure to degrade misfolded proteins activate UPR, which can upregulate the chaperones and ERAD components to increase protein fold capacity (8). ERAD facilitates removal of misfolded proteins from the ER for ubiquitin- proteasomal degradation (4). The suppressor/enhancer of lin-12-like (Sel1L)–HMG-CoA reductase degradation protein 1 (HRD1) complex is the most-conserved and best-characterized branch of ERAD. In recent studies, genetic deletion of several components of ERAD in specific cells of animals point out its indispensable physiological function. In this review, we highlight recent advances in understanding the endogenous substrates, the physiological role of Sel1L-HRD1 ERAD in metabolism.

## ERAD working mechanism

**Figure 1:**
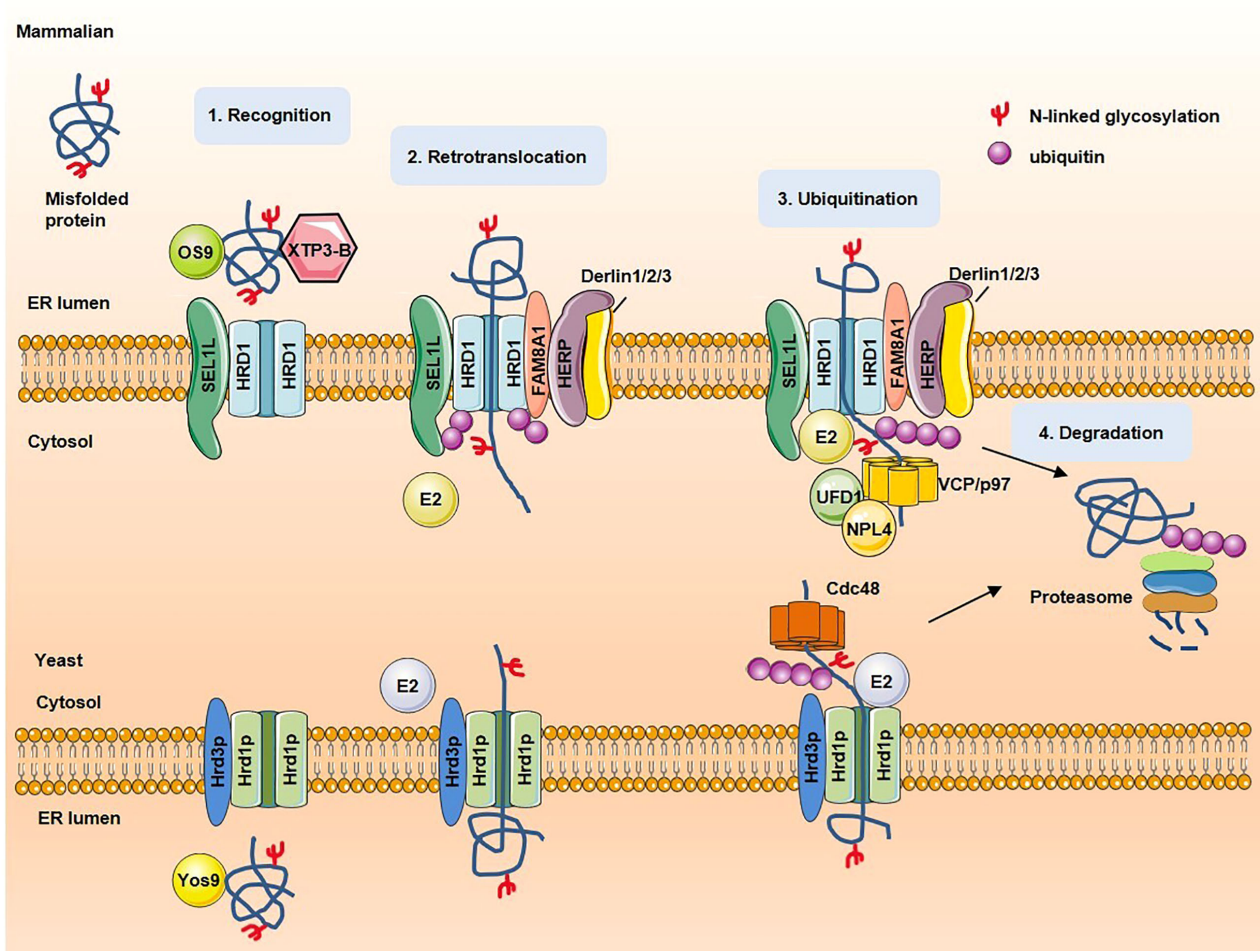
The steps and proteins involved in ERAD. In mammalian, misfolded proteins are recognized by OS9/XTP3-B and brought to ubiquitin ligase HRD1 complexes. Substrates are retrotranslacated trough channel formed by HRD1, then conjugated with polyubiquitin and extracted to cytosol for degradation by VCP–UFD1–NPL4 heterotrimer. In yeast, misfolded proteins are recognized by lectin Yos9 and Hrd3p and then brought to the ER membrane to interact with Hrd1p. On recognition, substrates are retrotranslocated into the cytosol through Hrd1p channel. Once exposed to the cytosolic face of the ER membrane, substrates are ubiquitinated. The cytosolic Cdc48 complex binds to ubiquitinated substrates and facilitates late stages of retrotranslocation by extracting substrates out of the ER membrane. Substrates are subsequently delivered to the proteasome for degradation.

ERAD substrates can be classified into four categories according to their degradation signal location: ERAD-L (lumen), ERAD-M (membrane), ERAD-C (cytosol) and ERAD-T (translocon). In yeasts, the best characterized ERAD pathways are mediated by the Hrd1p complex ((ERAD-L, ERAD-M, and ERAD-T) and Doa10p complex (ERAD-C, ERAD-M) (9, 10). In mammalian cells, several E3 ligases participate in ERAD, including HRD1, glycoprotein 78 (gp78), membrane-associated RING finger protein 6 (MARCH6) (11). In yeasts, ERAD-L substrates are recognized by lectin Yos9 and Hrd1p complex receptor Hrd3p and then brought to the ER membrane to interact with Hrd1p. ERAD-M/ERAD-C substrates are recognized by Doa10p complex. Substrates are then tagging with conjugation a polyubiquitin by Hrd1p and Hrd1p itself is also auto-ubiquitinated to recruit Cdc48p ATPase complex. Subsequently, ATP hydrolysis by Cdc48p releases substrate from ER to cytosol for degradation. Finally, the de-ubiquitinating enzyme (DUB) recruited and activated by the Cdc48p complex removes ubiquitin chains of Hrd1p (12). In mammalians, substrates are recognized by OS9/XTP3-B and HRD1 complex receptor Sel1L and brought to HRD1 complex. FAM8A1 increases the binding of HERP to HRD1, HERP facilitates Derlin1/2/3 recruitment and HRD1 oligomerization. Substrates are then conjugated with polyubiquitin and retrotranslocated to cytosol by VCP–UFD1–NPL4 heterotrimer (13, 14). **Figure 1** shows a schematic of the proposed mechanism for key processing steps in ERAD.

## ERAD controls protein quality and quantity

The proteins that fail to achieve their mature conformation due to genetic mutations, errors in transcription or translation or inefficient assembly into complexes are recognized by ERAD for quality control. Mutations in cystic fibrosis transmembrane conductance regulator (CFTR) gene are the cause of cystic fibrosis (15). The ΔF508 mutant of CFTR is retained in the ER and degraded efficiently by ERAD despite partial functionality (16). In yeasts, a Gly255Arg mutation of carboxypeptidase (CPY*) imports into the ER but never reaches the vacuole. The CPY* mutant is retained in the ER and rapidly degraded by ERAD (17, 18). More mutation proteins degraded by ERAD are listed in **Table 1** (19–28).

**Table 1:** Mutant proteins degraded by ERAD.

Substrates	E3 ligase	Species	Reference
CPY*	Hrd1p	Yeast	(19)
Ste6*	Doa10p	Yeast	(20)
Pexl 5Δ30	Doa10p	Yeast	(21)
NHK Al AT	HRD1	Mammalian	(22, 23)
Z-Al AT (E342K)	HRD1	Mammalian	(24)
Tyrosinase-C89R	HRD1	Mammalian	(25)
CFTRΔF508	Gp78/RNF5/RMA1/RNF185	Mammalian	(26–28)

In addition, ERAD also can recognize and degrade the properly folded proteins to control protein quantity. These substrates include ER-resident and secretory proteins, such as sterol synthesis enzyme (3-hydroxy-3-methylglutaryl-coenzyme A reductase, HMGCR) (29, 30), ER transmembrane sensor (Inositol-requiring enzyme 1α, IRE1α) (31). The discovery of more endogenous substrates will help to reveal the complex mechanism and physiological function of ERAD.

## Substrate retrotranslocation through Hrd1 channel

ERAD substrates must transport across the ER lipid bilayer to reach the cytoplasm for proteasomal degradation. Several candidates forming the ERAD channel have been proposed, including Sec61 (32, 33), Derlin family proteins (34, 35), and Hrd1 (12, 36, 37). In yeasts, a Hrd1 dimer associated with Hrd3 forms a channel which allows substrates to pass through the ER lipid bilayer. In this process, Der1 also intimately contacts retrotranslocating polypeptides. Usa1 associates with Hrd1, promoting its dimerization, and the cytosolic domain of Ubx2 recruits Cdc48, an AAA^+^−ATPase to the Hrd1 complex for retrotranslocation. The initial Hrd1 channel forming is triggered by autoubiquitination of Hrd1, binding of misfolded substrates to the Hrd1 further opens the channel, allowing for substrates translocation (38–41). De-ubiquitination of Hrd1 by DUB enzymes allows it to escape uncontrolled degradation and return to its inactive ground state. The cycles of autoubiquitination and de-ubiquitination of Hrd1 regulate ERAD to maintain ER homeostasis (42). Whether such a mechanism applies to the mammalian cells remains to be further studied.

## Physiological functions of ERAD in metabolism

**Figure 2:**
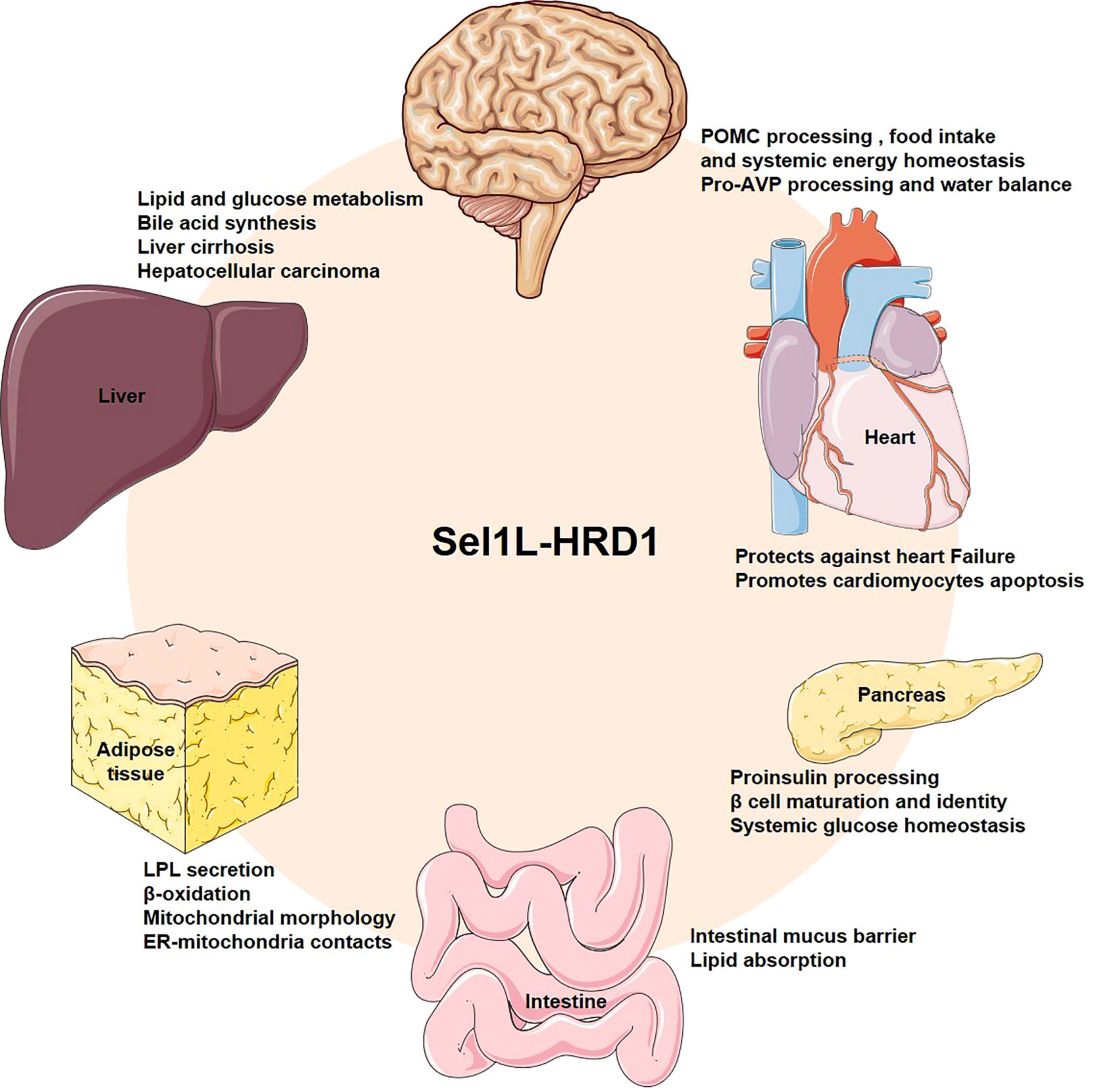
Overview the physiological roles of Sel1L-Hrd1 ERAD. Generation of cell type-specific deletion of ERAD related genes in mice have revealed the physiological roles of ERAD. Hrd1-Sel1L ERAD can coordinate metabolism in different specific cell types, such as hepatocytes, adipocytes, pancreatic β-cells, enterocytes, cardiomyocytes and neurons to maintain systemic metabolic homeostasis.

**Figure 3:**
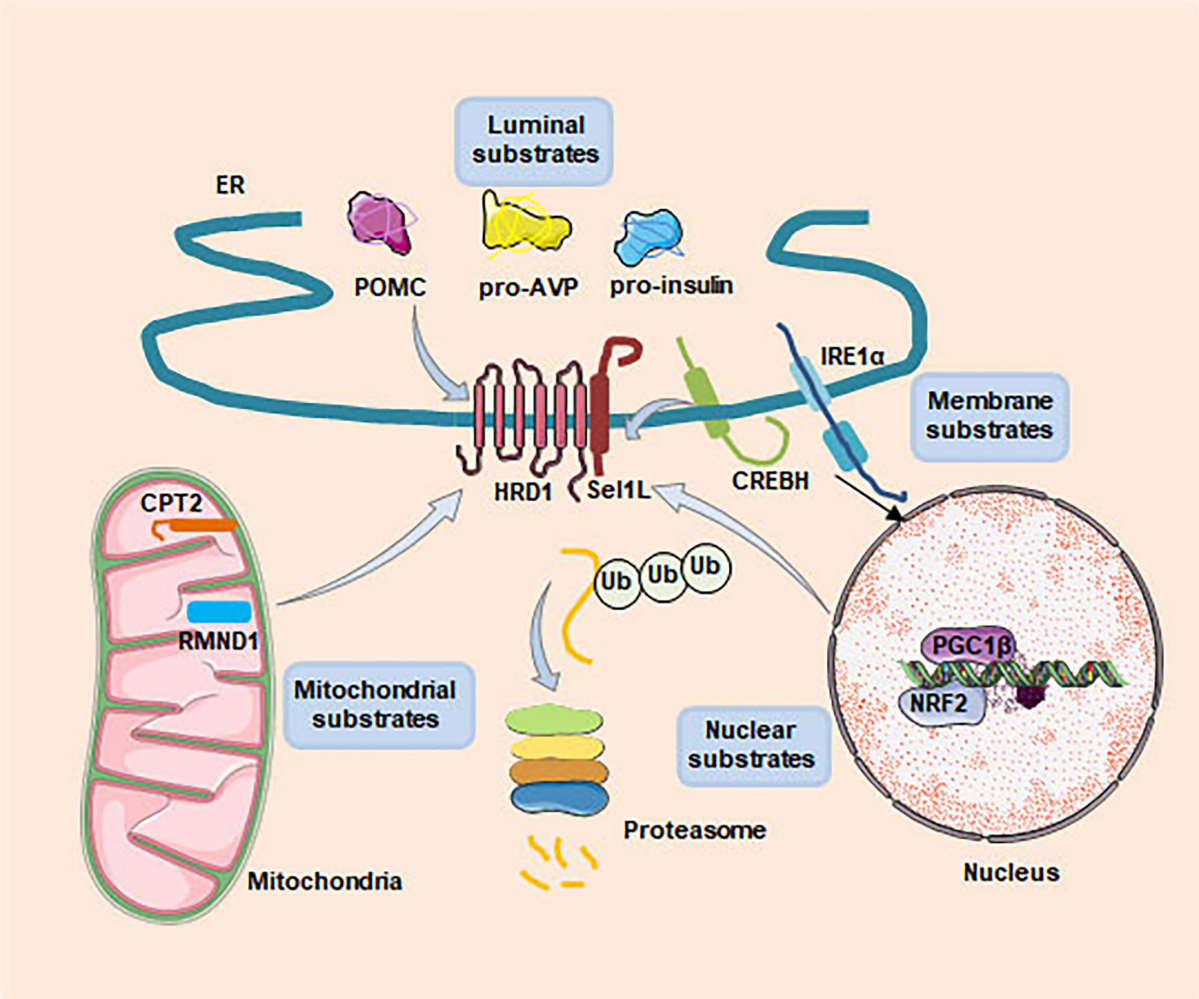
Recently identified substrates of Sel1L-Hrd1 ERAD. Four types of substrates, including ER luminal (POMC, pro-AVP and proinsulin), membrane (IRE1α and CREBH), nuclear (NRF2 and PGC1β) and mitochondrial (CPT2 and RMND1), have been identified as Sel1L-Hrd1 ERAD substrates. These studies have demenstrated that ERAD performs its physiological functions in a cellular and substrate-specific form.

Loss of key component of ERAD in mice, such as Hrd1, Sel1L, Derlin1 and Vcp, results in embryonic lethality, indicating that ERAD plays an important physiological role in embryonic development (43–46). In recent years, generation of cell type-specific deletion of ERAD related genes in mice have opened a door for us to understand the physiological functions of ERAD. These studies have revealed that ERAD performs its physiological functions in a cellular and substrate-specific form. Here, we summarize Hrd1-Sel1L ERAD regulates whole body metabolism by coordinating different specific cell types, such as in the hepatocytes, adipocytes, pancreatic β-cells, enterocytes, cardiomyocytes and neurons (**Figure 2**). Moreover, we also sum up a number of endogenous substrates for the Sel1L-Hrd1 complex (**Figure 3**, **Table 2**).

**Table 2:** Native folded substrates of ERAD, their subcellular localization and pathology.

Substrates	Subcellular localization	Cell type	Mouse model, phenotype and reference
Crebh	ER membrane, Nucleus	Hepatocytes	*Alb*-cre; *Sel1L* ^f/f^, growth retardation, female fertility, Elevated Fgf21 levels (47) *Alb*-cre; *Hrd1* ^f/f^, growth retardation, female infertility, diurnal circadian behavior disruption, elevated circulating Fgf21 (48) *Alb*-cre; *Hrd1* ^f/f^, increased FA oxidation, TG lipolysis, lipophagy, and gluconeogenesis, impaired circadian profiles of circulating TG, FA and glucose (49)
Entpd5 Cpt2 Rmnd1 Hsd17b4	ER lumen Mitochondira Mitochondria Peroxisome	Hepatocytes	*Alb*-cre/*Mx1*-cre; *Hrd1* ^f/f^, reduction of body weight, triglyceride, protect mice from HFD-induced obesity (50)
Acly	Cytosol	Hepatocytes	*db/db* mice injected with Hrd1 adenovirus, represses lipid synthesis, ameliorates systemic glycemic control and insulin sensitivity, attenuates steatosis (51)
Hnf1a Hnf4a	Nucleus Nucleus	Hepatocytes	*Sel1L* ^+/-^, increased serum total bile acid concentrations (52)
Nrf2	Nucleus	Hepatocytes	*Hrd1* ^f/f^ mice are injected with Cre viruses, Suppression of anti-oxidation pathway, liver cirrhosis (53)
Wnt5a	ER lumen, secreted	Hepatocytes	*Alb*-cre; *Sel1L* ^f/f^, *Alb*-cre; *Hrd1* ^f/f^, are prone to developing chemically induced liver tumors, exhibit excessive proliferation following injury (54)
Pgc1β	Nucleus	adipocytes	CAG-Cre-ER; *Hrd1* ^f/f^, CAG-Cre-ER; *Hrd1* ^f/f^; *ob*/*ob* or *db/db, Adiponectin*-cre; *Hrd1* ^f/f^, weight loss and lower accumulation of white adipose tissue (55)
SigmaR1	ER, cell membrane	adipocytes	*Adiponectin* or *UCP1*-cre; *Sel1L* ^f/f^, *Adiponectin* or *UCP1*-cre; *Hrd1* ^f/f^, cold sensitive and exhibit mitochondrial dysfunction, affect ER-mitochondria contacts and mitochondrial dynamics (56)
Proinsulin	ER lumen, secreted	Pancreatic β-cells	*RIP*-cre; *Sel1L* ^f/f^, *Ins1*-cre; *Sel1L* ^f/f^, reduced glucose-stimulated insulin secretion, hyperglycemia, glucose intolerance (57)
Tgfbr1	Cell membrane	Pancreatic β-cells	*Ins1*-cre; *Sel1L* ^f/f^, progressive hyperglycemia and glucose intolerance (58)
MafA	Nucleus	Pancreatic β-cells	*db/db* mice injected with AAV8-MIP-HRD1-GFP virus (59)
Ire1α	ER membrane	Enterocytes	*Villin*-cre; *Sel1L* ^f/f^, susceptible to experimental colitis and inflammation-associated dysbiosis (60)
Gpat3 Dgat2 Mogat2	ER membrane	Enterocytes	*Villin*-cre; *Aida* ^f/f^, HFD-induced obesity, increased fatty acid re-esterification and lipid absorption in intestine, postprandial hypertriglyceridemia (61)
Pomc	ER lumen, secreted	POMC neurons	*POMC*-cre; *Sel1L* ^f/f^, age-associated obesity and hyperleptinemia, hyperphagia, impaired leptin sensitivity (62)
Pro-Avp	ER lumen, secreted	AVP-producing neurons	*AVP*-promoter driving cre; *Sel1L* ^f/f^, central diabetes insipidus, intracellular ER retention of proAVP (63)

## In hepatocytes

As we all know, liver is a key organ which controls lipogenesis, gluconeogenesis and cholesterol metabolism. The expression level of Sel1L-Hrd1 in hepatocytes varies in response to metabolic signals during growth and fasting-feeding (47, 48, 50). Liver-specific deletion of *Hrd1* or *Sel1L* in mice leads to growth retardation, reduced female fertility and elevated circulating fibroblast growth factor 21(Fgf21) (47, 48, 50, 64). Further mechanistic studies have shown that Sel1L-Hrd1 controls Fgf21 transcription by facilitating the well-known transcriptional factor of Fgf21, cAMP-responsive element-binding protein 3-like protein 3 (Creb3l3, also known as Crebh) for ubiquitin-proteasomal degradation (47, 48). Hepatocyte-specific *Hrd1* knockout mice also exhibit decreased the body weight, lipid and glucose levels, and resistance to HFD-induced obesity and NAFLD. Hrd1 programs hepatic energy metabolism by regulating Entpd5, Cpt2, Rmnd1, and Hsd17b4 protein levels. In addition, Hrd1 relieves NAFLD in *db/db* mice by degrading ATP citrate lyase (Acly), which can catalyze the cleavage of citrate into oxaloacetate and acetyl-CoA, the latter serving as common substrate for *de novo* cholesterol and fatty acid synthesis (51).

The expression of Sel1l-Hrd1 in liver fluctuates rhythmically with circadian rhythm and is regulated by Crebh, peroxisome proliferator-activated receptor α (Pparα) and the core clock oscillator Bmal1. *Hrd1* liver-specific knockout mice exhibit increased expression of the genes involved in fatty acid (FA) oxidation, triglyceride (65) lipolysis, lipophagy, and gluconeogenesis and impaired circadian profiles of circulating TG, FA and glucose. Hrd1 promotes Crebh circadian rhythmic polyubiquitination and degradation. Hrd1/Sel1L-Crebh/Pparα regulatory axis regulates the genes involved in hepatic FA oxidation, TG lipolysis, lipophagy, and gluconeogenesis to maintain circadian rhythmic levels of circulating TG, FA, beta-hydroxybutyrate and glucose in mice (49).

In a recent study, researchers conducted a genome-wide association study to identify loci that accounted for liver-related phenotypes between C57BL/6J and A/J mice fed a Paigen diet and identified Sel1L as a candidate gene for variation in serum total bile acids concentration. Further studies showed that this risk allele locally reduced SEL1L expression in the liver and down-regulated pathways associated with hepatocyte nuclear factor 1 homeobox A (HNF1A) and hepatocyte nuclear factor 4A (HNF4A), which are known to be modifier factors of bile acid transporters and metabolic traits. Depletion of SEL1L in HepG2 cells resulted in reduced HNF1A and HNF4A protein levels and increased bile acids in culture media. Dogs with *SEL1L* ^S658P/S658P^ mutation and *Sel1L* ^+/-^ mice fed a Paigen diet exhibit significantly increased serum total bile acid concentrations, providing evidences for the involvement of SEL1L in bile acid metabolism (52). Future studies will be required to demonstrate the relationship between Sel1L, Hrd1 and serum bile acid levels by using liver-specific Sel1L or Hrd1 deficient mice.

Liver cirrhosis is a common chronic progressive liver disease caused by the long-term or repeated damage of one or more etiology. The Xbp1–Hrd1 arm is upregulated and the nuclear factor erythroid 2-related factor 2 (Nrf2) antioxidative pathway is downregulated in human or CCl4-treated mice cirrhotic liver tissues. Mechanistically, UPR is activated and transcriptionally up-regulated Hrd1 through the XBP1–Hrd1 arm in cirrhotic livers. Hrd1 promotes the ubiquitin-proteasomal degradation of Nrf2 to attenuate Nrf2 mediated protection function in liver cirrhosis. Moreover, inhibition of Hrd1with small molecule inhibitor LS-102 alleviate liver cirrhosis (53). Pharmacological inhibition of Hrd1 might be a new strategy to alleviated liver injury and cirrhosis.

Obesity, T2DM and NAFLD are the major hepatocellular carcinoma pathogenic factors. Mice specific deletion of *Sel1L* or *Hrd1* in liver are more susceptible to diethylnitrosamine (DEN)/high fat diet (HFD)-induced tumors. Proteomics analysis identified Wnt5 is a substrate of ERAD linking Sel1L-Hrd1 to hepatocyte hyperproliferation and hepatic carcinoma (54).

## In adipocytes

Adipocytes regulate energy metabolism through storing energy in the form of lipid droplets in white adipocytes and consuming energy in brown and beige adipocytes. Obesity is characterized by excessive accumulation of fat and disruption of lipid metabolic balance. Adipose tissue is also an endocrine organ to secret adipocytokines, including leptin and adiponectin. Adipocyte-specific deficiency of *Sel1L* protects mice from diet-induced obesity and predisposes mice to postprandial hypertriglyceridemia. Further studies reveal that Sel1l facilitates LPL secretion from ER to control systemic lipid partitioning (66).

Moreover, loss of *Hrd1* in post-neonatal mice or adipose-specific deletion of *Hrd1* results in a decrease in body weight and fat mass. Microarray analysis in white adipose tissues shows that genes involved in β oxidation and mitochondria biogenesis is increased in *Hrd1* deficient mice. Hrd1 targets thermogenic coactivator peroxisome proliferator activated receptor coactivator β (PGC1β) for degradation to regulate energy expenditure (55). Mice with *Sel1L-Hrd1* ERAD deficiency in brown adipocytes exhibit impaired mitochondrial function and thermogenic response. Sel1L-Hrd1 ERAD regulates mitochondrial morphology and ER-mitochondria contacts via controlling SigmaR1 protein turnover (56). Although more research is needed to elucidate how Sel1l-Hrd1 ERAD coordinates systemic lipid metabolism in different tissues, ERAD in adipose tissue may be an effective target for treatment of obesity.

## In pancreatic β-cells

Pancreatic β-cells are essential to maintain systemic glucose homeostasis and failure to compensate for insulin resistance is a key mechanism leading to T2DM (67, 68). Several studies have demonstrated that ERAD plays a critical role in glucose-stimulated insulin secretion in pancreatic β-cells. Haploid insufficiency of *Sel1L* in mice is susceptible to HFD-induced hyperglycemia and impairs glucose stimulated insulin secretion (69). Pancreatic β-cell specific *Sel1L* knockout mice show impaired proinsulin processing in the endoplasmic reticulum and susceptible to diabetes mellitus. Sel1L-Hrd1 facilitates proinsulin for proteasomal degradation to sustain pancreatic β-cells function. ERAD-deficiency in pancreatic β-cells also influences Ca^2+^ signaling and mitochondrial function (57, 70). In a recent study, single cell sequencing analysis reveals that genes involved in autophagy are upregulated in *Sel1L* deficient β-cells. In the absence of Sel1L-Hrd1, autophagy is activated to clear the retained proinsulin. Moreover, deletion of both *Sel1L* and *Atg7* in β-cells leads to diabetes in mice shortly after weaning, with premature death by ~11 weeks of age, associated with marked ER retention of proinsulin and β-cell loss. SEL1L-HRD1 ERAD and autophagy synergistically regulate β cell survival and systemic glucose homeostasis. Mechanistically, RTN3, an ER-phagy adaptor, mediates ER-phagy to degrade misfold proinsulin in *Sel1L* deficient islet. ERAD and autophagy remodel organellar network and determine the ER architecture of β cells (71). Another study reveals that depletion of Sel1L does not lead to β cell loss, but rather impaired β cell maturation and identity. Sel1L-Hrd1 ERAD controls β cell identity via targeting TGF-β receptor 1 for degradation (58).

In genetic or HFD induced diabetic mice, the Hrd1 expression of pancreatic β cells is elevated. Similar results are observed in the islets isolated from T2D humans. β-cell-specific knockdown Hrd1 improves hyperglycemia and glucose intolerance in diabetic mice. MafA, an essential β-cell-specific transcriptional factor to activate insulin expression is identified as a Hrd1 interacting protein by mass spectrometric. Hrd1 promotes MafA retrotranslocation to cytosol for degradation and weaken its transcriptional function in the nucleus (59).

In general, Sel1L-Hrd1 is essential to maintain pancreatic β cells function under normal physiological condition. In the islets of diabetic mice or human, oxidative stress and ER stress is elevated dramatically leading to excessive Hrd1 expression (72, 73). These results suggest that the expression level and substrates of Hrd1 in physiological and pathological conditions may differ. The underlying mechanism of Sel1L-Hrd1 switching under physiological and pathological conditions needs to be further investigated.

## In enterocytes

Enterocytes are not only responsible for nutrient absorption, but also can form a physical barrier to prevent bacteria from invading intestinal mucosa. Enterocyte-specific *Sel1L* knockout mice are susceptible to dextran sodium sulfate induced colitis and *Toxoplasma gondii*–induced ileitis (31, 60). In Sel1L deficient enterocytes, Ire1α protein level is elevated by 15-fold, while its mRNA level is not altered. Sel1L-Hrd1 mediated Ire1α degradation is regulated by ER stress in a BiP- dependent manner. ER stress increases the accumulation of misfolded proteins in the ER, which competes with Ire1α for interacting with ERAD and attenuates Sel1L-Hrd1-mediated Ire1α ubiquitination and degradation. ER stress also triggers BiP- Ire1α dissociation and dimer-/oligomerization of Ire1α, leading to Ire1α dissociation from the ERAD complex and protein stabilization (31).

Furthermore, Aida, an Axin interactor (74) can selectively mediate Hrd1 for degradation of triglyceride synthesis enzymes in enterocytes. When mice are gavaged with olive oil and ERAD inhibitor Eeyarestatin I, the protein level of glycerol-3-phosphate acyltransferase 3 (Gpat3), monoacylglycerol o-acyltransferase 2 (Mogat2), and diacylglycerol o-acyltransferase 2 (Dgat2) are significantly elevated in enterocytes. Meanwhile, Eeyarestatin I pre-treatment significantly increases serum triglyceride to similar extent in untreated Aida knockout mice (61). These results suggest Aida-mediated HRD1 negatively regulates dietary fat absorption via targeting Gpat3, Mogat2, Dgat2. Using enterocytes-specific knockout HRD1 mice to further reveal the role of ERAD in fat absorption, postprandial hypertriglyceridemia and obesity is required in future study.

## In cardiomyocytes

Cardiovascular diseases caused by metabolic disorders, hypoxia, and inflammation increase the protein folding load of the ER, consequently triggering ER stress (75, 76). ERAD removes the misfolded proteins from ER lumen to counteract the ER stress. Knockdown Hrd1 in the cardiomyocytes of mice via AAV9-sh-Hrd1 exacerbates pathological cardiac hypertrophy when subjected to trans-aortic constriction (TAC) surgery and Hrd1 overexpression protects cardiac function. Depletion of Hrd1 in cultured cardiomyocytes increased the ER stress makers, Grp94 and Grp78, thereby activating apoptosis. These results demonstrate that Hrd1 plays a key role in the adaptation of cardiac myocytes to ER protein misfolding (77). Moreover, another study finds the protein level of Hrd1 is increased in cardiac tissues of acute myocardial infarction rats and hypoxia-induced cardiomyocytes. Depletion of Hrd1 in cardiomyocytes inhibits apoptosis induced by hypoxia. Further research confirms that Hrd1 promotes insulin like growth factor 1 receptor (IGF-1R) ubiquitination for degradation via Semaphorin-3A. Hrd1 negatively regulates IGF-1R to promote cardiomyocytes apoptosis and accentuate acute myocardial infarction (78). Collectively, in the TAC surgery mediated cardiac hypertrophy, Hrd1 is adaptive to pressure overload and inhibits cardiomyocytes apoptosis, but in the hypoxia state, Hrd1 promotes cardiomyocytes apoptosis and aggravate acute myocardial infarction. Future studies using cardiomyocyte-specific Sel1L or Hrd1 deficient mice will be required to establish a causal relationship between Sel1L-Hrd1 ERAD and different cardiac diseases, and to find cardiomyocyte-specific substrates of ERAD.

## In neurons

Pro-opiomelanocortin (POMC) neurons are located at the hypothalamic arcuate nucleus (ARC) region and release several neuropeptides derived from POMC, which signals satiety to regulate food intake and systemic energy homeostasis (79–82). POMC undergoes tissue-specific proteolysis to generate biologically active peptides and hormones, including adrenocorticotropin (ACTH), α-, β-, γ- melanocyte stimulating hormone (MSH) and β-endorphin. Processing of POMC into mature peptides is accomplished by a series of enzymatic steps that occur in the ER, Golgi and secretory granules. Evidences from animal studies and human genetic analysis have implicated that POMC-derived peptides are associated with obesity (80, 83–92). Hrd1 is highly expressed in ARC and responsive to refeeding behavior. Mice specific deletion of *Sel1L* in POMC neurons develop age-associated obesity and hyperleptinemia. *Sel1L* ^POMC^ knockout mice exhibit increased food intake and impaired leptin sensitivity because of intracellular retention of POMC. Sel1L-Hrd1 ERAD targets unfolded or misfolded POMC for proteasomal degradation and ensures POMC processing to bioactive peptides (62). A mutation of POMC at position 28 (cysteine-to-phenylalanine, POMC-C28F) has been identified in patients with early-onset obesity (92). POMC-C28F is misfolded and forms aggregates via intermolecular C50-mediated disulfide bonds, thus evading Sel1L-HRD1 ERAD for quality control (62).

Arginine-vasopressin (AVP), a neuropeptide, is synthesized in AVP-producing neurons and regulates water balance (93–95). Heterozygosity for an AVP mutation leads to familial neurohypophyseal diabetes insipidus (96). Hrd1 is highly enriched in AVP neurons and increased during water deprivation. Mice with global or AVP neuron-specific deletion of *Sel1L* develop central diabetes insipidus and result in intracellular ER retention of pro-AVP. Mechanistically, pro-AVP is an endogenous substrate of Sel1L-Hrd1 ERAD. Ablation of Sel1L-Hrd1 ERAD causes pro-AVP aggregation in a disulfide bond–dependent manner (63). Future studies to research the physiological roles and substrates of ERAD in other specific neurons of brain are required.

## Conclusions and future perspectives

Although ERAD is a well-known protein quality and quantity control system in the ER, its physiological functions remain poorly understood due to embryonic lethality in ERAD gene knockout mice. Recently, Cell type-specific knockout of ERAD components in mice reveals that ERAD are crucial for maintaining metabolic homeostasis in a substrate-dependent manner (**Table 1**, **Figure 1**). Changes in physiological status (fasted and refed, growth, circadian rhythm, water deprivation and ER stress) can induce HRD1 expression in response to cellular stress in specific cell types (47, 49, 50, 63, 97). As a critical metabolic regulator, whether the expression of HRD1 is altered in human metabolic disorders needs further research. Moreover, there is still lack of a measurable indicator to quantify the activity of ERAD in physiological and pathological states. Achieving this may also make it possible for HRD1 to be a target for the treatment of metabolic disorders in humans.

The endogenous ERAD substrates are not only located in the ER lumen and membrane, but also in other cellular substructures such as mitochondria and nucleus (**Figure 2**). Examples include the mitochondrial proteins Cpt2 and Rmnd1, which is required for β-oxidation and protein translation, protect mice from HFD-induced obesity, the nucleus protein Pgc1β, involved in fat oxidation and non-oxidative glucose metabolism and in the regulation of mitochondrial biogenesis and energy expenditure (50, 55). However, the underlying mechanism of how Hrd1 recognizes these non-ER localized substrates and whether this process depends on other adaptors remains unclear.

Metabolism and inflammation are intertwined to regulate vital activity in the broadest manner. Chronic metabolic inflammation in multiple organs include liver, adipose tissue, pancreas and brain is implicated in metabolic disorders (98, 99). In the context of obesity and associated metabolic disorders, chronic inflammation leads to recruit of pro-inflammatory M1 macrophages into the adipose tissue stroma to secret tumor necrosis factor (TNF) that induces insulin resistance (100, 101). However, future studies to reveal whether ERAD is involved in metaflammation to contribute to metabolic disorders are required.

ERAD, UPR and autophagy are the three main protein quality control systems and inextricably linked each other. Although several studies have revealed the crosstalk among the three systems within the cell (8), how and when they perform complementary or different functions remain poorly known. Understanding the crosstalk mechanisms of ERAD, UPR and autophagy in both physiological and pathological conditions should contribute to the development of therapies for treating metabolic diseases.

## Author contributions

Manuscript design, HL and XZ. Literature review, QJ, CBS, CYS, JX, YC, and XF. Drafting of manuscript, HL. Critical revision of manuscript, HL and XZ. Funding acquisition, HL. Final approval of manuscript, HL, QJ, CBS, CYS, JX, YC, XF, and XZ. All authors contributed to the article and approved the submitted version.
